# The Emerging Role of Super-enhancers as Therapeutic Targets in The Digestive System Tumors

**DOI:** 10.7150/ijbs.78535

**Published:** 2023-01-22

**Authors:** Xiang-Ping Li, Jian Qu, Xin-Qi Teng, Hai-Hui Zhuang, Ying-Huan Dai, Zhi Yang, Qiang Qu

**Affiliations:** 1 Department of Pharmacy, Xiangya Hospital, Central South University, Changsha 410007, PR China.; 2 Institute for Rational and Safe Medication Practices, National Clinical Research Center for Geriatric Disorders, Xiangya Hospital, Central South University, Changsha 410007, PR China.; 3 Department of Pharmacy, the Second Xiangya Hospital, Central South University; Institute of Clinical Pharmacy, Central South University, Changsha 410011, PR China.; 4 Hunan key laboratory of the research and development of novel pharmaceutical preparations, Changsha Medical University, Changsha, 410219, PR China.; 5 Department of Pathology, the Second Xiangya Hospital, Central South University, Changsha 410011, PR China.; 6 Department of Colorectal and Anal Surgery, Hepatobiliary and Enteric Surgery Research Center, Xiangya Hospital, Central South University, Changsha 410007, PR China.

**Keywords:** digestive system tumors, super-enhancer, tumor progression, non-coding RNAs, therapeutic targets.

## Abstract

Digestive system tumors include malignancies of the stomach, pancreas, colon, rectum, and the esophagus, and are associated with high morbidity and mortality. Aberrant epigenetic modifications play a vital role in the progression of digestive system tumors. The aberrant transcription of key oncogenes is driven by super-enhancers (SEs), which are characterized by large clusters of enhancers with significantly high density of transcription factors, cofactors, and epigenetic modulatory proteins. The SEs consist of critical epigenetic regulatory elements, which modulate the biological characteristics of digestive system tumors including tumor cell identity and differentiation, tumorigenesis, environmental response, immune response, and chemotherapeutic resistance. The core transcription regulatory loop of the digestive system tumors is complex and a high density of transcription regulatory complexes in the SEs and the crosstalk between SEs and the noncoding RNAs. In this review, we summarized the known characteristics and functions of the SEs in the digestive system tumors. Furthermore, we discuss the oncogenic roles and regulatory mechanisms of SEs in the digestive system tumors. We highlight the role of SE-driven genes, enhancer RNAs (eRNAs), lncRNAs, and miRNAs in the digestive system tumor growth and progression. Finally, we discuss clinical significance of the CRISPR-Cas9 gene editing system and inhibitors of SE-related proteins such as BET and CDK7 as potential cancer therapeutics.

## Introduction

Digestive system tumors including esophageal cancer, colorectal cancer, gastric cancer, pancreatic cancer, hepatic cancer, cholangiocarcinoma, and gallbladder carcinoma are associated with significantly high mortality and morbidity rates 1. The multistep transformation of normal cells into malignant cells involves genetic and epigenetic alterations that promote the aberrant expression of critical oncogenes and tumor suppressor genes 1, 2. Epigenetic modifications alter cellular plasticity, differentiation, and reprogramming without changing the primary DNA sequence or the genetic code of organisms 3. Aberrant changes in epigenetic mechanisms regulating DNA methylation, histone methylation and acetylation, expression of noncoding RNAs, and mRNA methylation are associated with the initiation, growth, and progression of digestive system tumors 2, 4.

Previous studies have reported the role of super-enhancers (SEs) in digestive system tumors. In the post-genomic era, the cancer research is focused on the dysregulation of transcriptional dysregulation mediated by epigenetic modifications in the enhancer, SE, and gene promoter regions of key tumor suppressor and tumor-promoting genes 5, 6. SEs are defined as large clusters of active enhancers in large clusters that are enriched with high levels of transcription factors (TFs), master co-factors, mediator complexes, RNA polymerase II (RNA pol II), and epigenetic modifications 5, 7. SEs regulate biological processes such as cell cycle, oncogenesis, cellular differentiation, immune response, and drug resistance 5-8. In this review, we summarized the transcriptional regulatory mechanisms, therapeutic targets, and oncogenes associated with SEs in the digestive system tumors.

## Overview of super-enhancers

### Definition and properties of enhancers and super-enhancers

**Figure 1:**
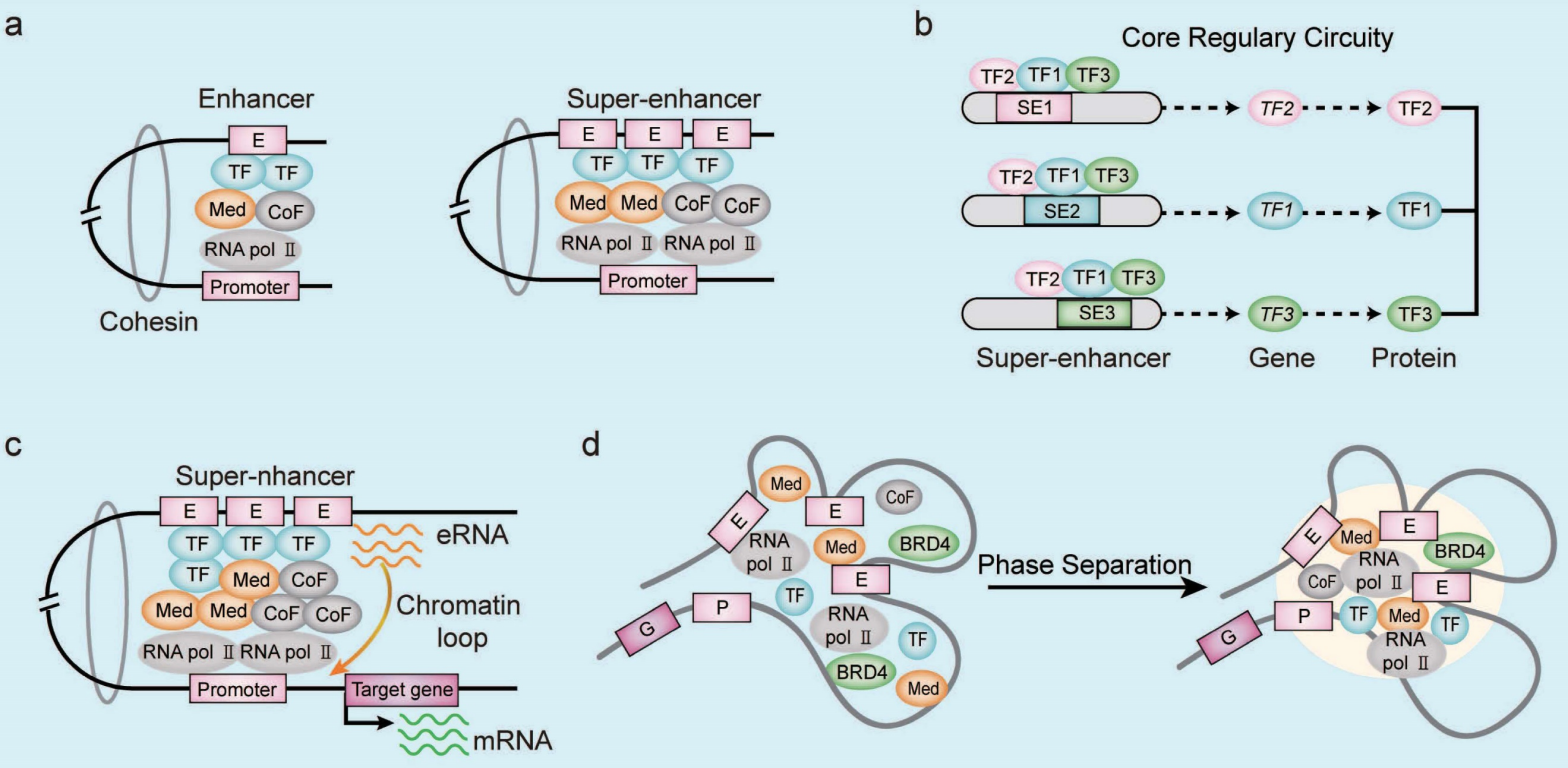
** Characteristics of SEs. (A)** Compared with the typical enhancers, super-enhancer regions are occupied by a higher density of transcription-related factors including transcription factors (TFs), co-activators, mediators, and RNA pol II complexes. **(B)** The core transcriptional circuit consists of several auto-regulated TFs and TF-related SEs, which are assembled into interconnected loops to coordinate the expression of cell identity- and tumor-related genes. **(C)** The enhancer RNAs (eRNAs) generated by the transcription of SE regions mediate chromatin looping between the SE and gene promoter sequences. The eRNAs induce formation and stabilization of the chromatin loop between SE and gene promoters by recruiting RNA Pol II, co-factors, and mediators. The eRNAs also assist in the promoter-enhancer circularization through cohesins. **(D)** The phase separation model of SE activation demosntrates high-density interactions between transcriptional regulatory factors that form distinct multi-molecular complexes in the SE region and drive the transcription of SE-associated genes. Note: E, enhancer; P, promoter; G, target gene; TF, transcription factor; CoF, cofactor; Med, mediator.

Enhancers are regulatory DNA sequences that enhance target gene transcription by modulating the gene promoter activity through specific TFs in the mammalian cells 9. Enhancers contain conserved DNA binding sites for the TFs, RNA polymerase, and co-activators 10. Functional enhancers are also characterized by specific epigenetic modifications such as histone H3 lysine4 methylation (H3K4me1), histone H3 lysine27 acetylation (H3K27ac), and others 11. The SE regions were first identified by chromatin immunoprecipitation-coupled sequencing (ChIP-seq) analysis of the mediator complex in the mouse embryonic stem cells (ESCs) 5. The SE regions span dozens of kilobases compared to the typical enhancer (TE) regions, which span only a few dozen base pairs 5. The SEs are enriched with a higher density of TFs, co-factors, and enhancer-associated epigenetic modifications compared to the TEs **(Figure 1A)**. Therefore, SEs drive stronger transcriptional activity than the TEs 5, 12. Furthermore, transcriptional coactivators such as cyclin-dependent kinase 7 (CDK7) and bromodomain protein 4 (BRD4) are enriched in the SE regions 13. SE-driven gene transcription involves cooperative activities of the constituent enhancers. Therefore, deletion of the constituent enhancers significantly alters the SE-driven gene transcription 14, 15. Cohesins are proteins that reduce the spatial distance between the enhancer region and the gene promoter elements 16. The transcription of SE-driven genes is highly sensitive to disruptions in the cooperative interactions between various SE-related factors because the three-dimensional chromatin architecture of the SE region is altered 16, 17.

The SEs are enriched in genomic regions that harbor disease-associated genetic variants and are potentially diagnosis and therapeutic targets. Several SEs involved in human diseases have been characterized by ChIP-seq 18, chromosome conformation capture 19, and DNase I coupled to high-throughput sequencing (DNase-seq) techniques 20.

### Biological functions of SEs

SEs are powerful cis-regulatory elements that define cell identity and modulate the maintenance and differentiation of stem cells by regulating the expression levels of pluripotency genes such as OCT4, SOX2, and NANOG 7, 21. Furthermore, the Hippo-YAP signaling pathway modulates the lineage differentiation of mESCs by regulating the formation of SEs 22. Aberrant SE-related transcription in the cancer cells is associated with increased proportion of cancer stem cells, tumor recurrence, drug resistance, and metastasis 23.

Immunotherapy has emerged as a promising cancer treatment with high efficacy. However, only a subset of patients responed to immunotherapy whereas others show minimal or no response because of variations in the complex tumor immune microenvironment 24. The immune response of digestive system tumors is also regulated by SEs 25. A Recent study showed that SEs promote chemoresistance of tumors by aberrant transcription of genes involved in chemoresistance 26. Additionally, SEs regulate the initiation and maintenance of chemoresistance in multiple tumors 6, 27. Furthermore, the sensitivity of chemotherapy can be restored by small molecule epigenetic inhibitors of SEs 6.

### Characteristics of SEs

The master TFs in the SE region form an interconnected core regulatory circuits, which orchestrates the transcriptional programs in both normal and malignant cells **(Figure 1B)** 28. The transcriptional program in the human ESCs involves collaboration between OCT4, SOX2, and NANOG, which form the core regulatory circuitry in the SE region with auto-regulatory and feed-forward loops 29. Similarly, ELF3, EHF, and TGIF1 form the interconnected core regulatory circuitry in the SE region of lung adenocarcinoma cells and promote transcription of the three master TFs and their related genes in tandem 30.

SEs also indirectly regulate the biological functions of cancer cells by modulating the expression levels of noncoding RNAs (ncRNAs) 31, including microRNAs (miRNAs) 32, long noncoding RNAs (lncRNAs) 33, and circular RNAs (circRNAs) 34. For example, knockdown of SE-associated lncRNA CCAT1-L significantly decreasing the *MYC* transcript levels by reducing the long-range interactions between *MYC* promoter and its enhancers 35. Enhancer RNAs (eRNAs) are ncRNAs that are transcribed from the DNA sequences in the SE regions and are involved in the regulation of gene expression, RNA splicing, translation, and epigenetic mechanisms 34, 36. The eRNAs regulate initiation, proliferation, apoptosis, migration, adhesion, drug resistance, and immune response of multiple tumors 37, 38. Moreover, eRNAs promote transcription by enhancing the formation of SE-gene promoter loops **(Figure 1C)** 39. Gene expression is regulated by the phase-separated multi-molecular assemblies of transcriptional protein complexes in the SE region 17. In the liquid-liquid phase separation (LLPS) model, SEs display a transcriptional bursting pattern by forming chromatin loops with their target gene promoters** (Figure 1D)** 40. Transcriptional co-activators BRD4 and MED1 are enriched in the SEs and form phase-separated droplets, which contributes to the compartmentalization and concentration of the transcriptional machinery from the nuclear extracts at the SEs 41. The intrinsically disordered regions (IDRs) associated with the SE-enriched transcriptional co-activators play an important role in the process of LLPS through self-associating electrostatic, polar, and hydrophobic interactions 40, 42, 43. For example, LLPS of NUP98-HOXA9 promotes transcriptional activation of the tumor genes by forming a broad super-enhancer-like binding patterns 44.

## Oncogenic roles and regulatory mechanisms of SEs in the digestive system tumors

**Figure 2:**
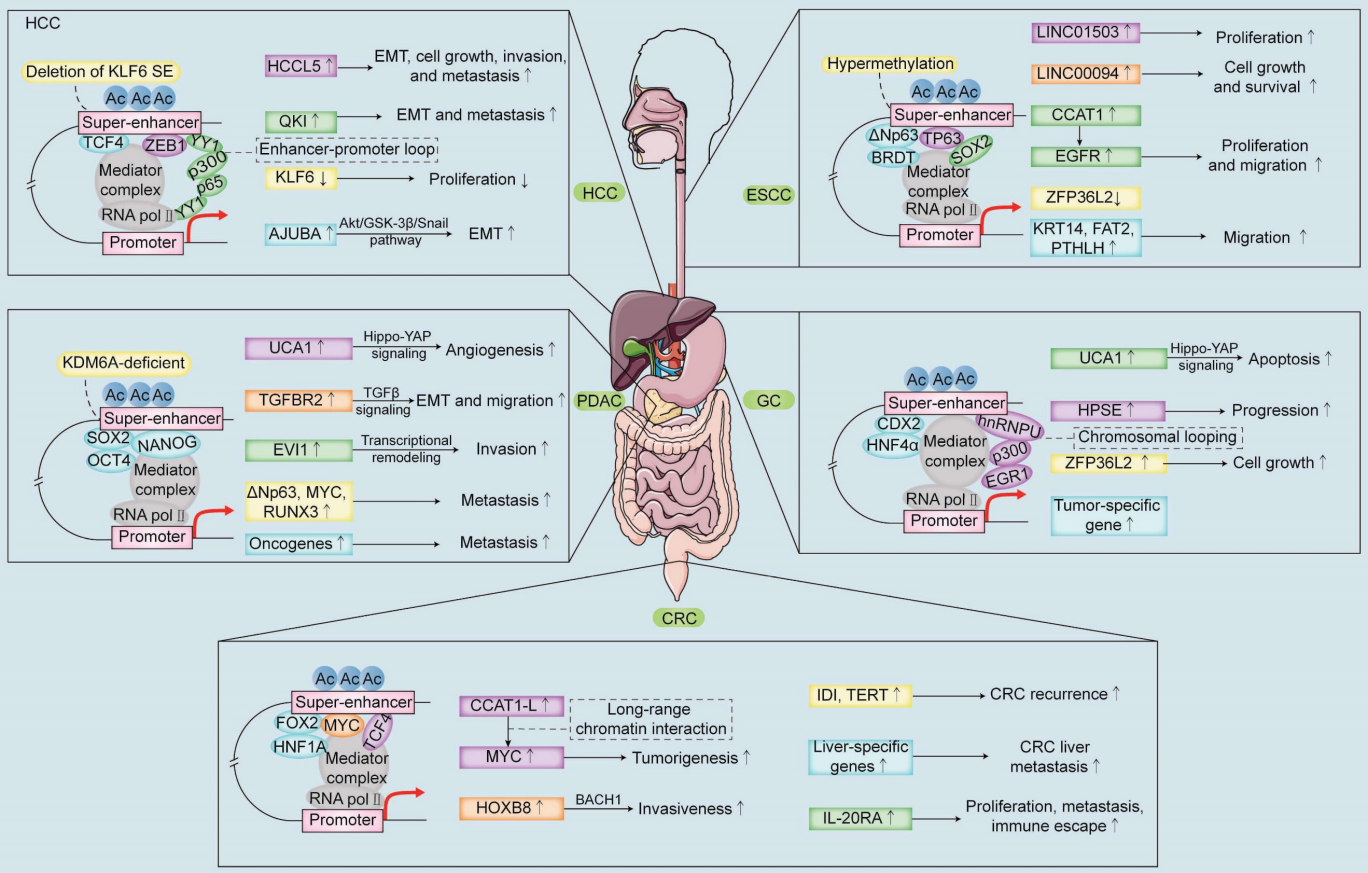
** Schematic representation of the mechanisms and biological functions of SEs in the five main solid tumors of the digestive system.** SE-related TFs and SE-target gene promoter loops regulate the expression of oncogenes or tumor suppressor genes and promote malignant proliferation, migration, invasion, metastasis, and recurrence of the digestive system tumors. Note: ESCC, esophageal squamous cell carcinoma; GC, gastric cancer; CRC, colorectal cancer; HCC, hepatocellular carcinoma; PDAC, pancreatic ductal adenocarcinoma.

SEs play a critical role in the development of multiple digestive system tumors (**Figure 2**). Genome-wide profiling of tumor tissues identified SE-associated oncogenes, *PHF19* and *TBC1D16* 45. Silencing of SE-related oncogenes significantly inhibit the tumor progression 45. SEs activate several oncogenes and signaling pathways in esophageal adenocarcinoma (EAC) 46. Four EAC-specific master regulatory transcription factors, ELF3, KLF5, GATA6, and EHF form interconnected regulatory loops, which co-operatively activate the expression levels of all the four TFs 47. These master TFs occupy most EAC-related SEs and promote the survival and proliferation of EAC cells through cooperative transcription of the EAC-related genes 46. In the following section, we discuss the oncogenic roles of SEs and the underlying mechanisms that regulate the biological processes in different digestive system tumors **(Table 1)**.

### SE participate in the transcription reprogramming of oncogenes

SEs demonstrate stronger transcriptional activity than the TEs 5. Cancer-related SEs promote malignant tumor progression by increasing the transcription levels of the oncogenes. For example, binding of* MYC* to the active elements in the SE region drives overexpression of* HOXB8* during colorectal cancer (CRC) progression 48. In some case, reorganization of SEs due to chromosomal rearrangements results in enhancer hijacking and is associated with the activation of oncogenes. In CRC, *IGF2* is a target of enhancer hijacking and is activated by the formation of a 3D contact domain that involves tandem-duplicated IGF2 and the lineage-specific SE 49. In several cases, SE-driven transcription is associated with tumor malignancy phenotypes. For example, SE-driven *EVI1* is a key promoter of oncogenic transcription remodeling and is associated with the aggressive malignant phenotype in pancreatic ductal adenocarcinoma (PDAC) 50. Similarly, SE-driven *TGFBR2* transcription promotes pancreatic cancer growth and progression via TGF-β signaling 51, 52. SE-mediated transcriptional activation regulates expression levels of several oncogenes. The overexpression of *ZFP36L2* promotes growth and malignancy of gastric cancer (GC) cells, and its transcription levels are regulated by a tandem duplication in the SE region 53. Interleukin-6 (IL-6) is overexpressed in pancreatic cancer. Bao et al. demonstrated that genetic deletion of IL6-SEa or treatment with two inhibitors of the SE-related BET protein, JQ-1 and I-BET762, significantly reduced the IL-6 expression levels in multiple cancer cells 54.

Cancer cells adapt to the dynamic tumor microenvironment through transcriptional reprogramming, which is significantly associated with tumor metastasis and recurrence. In ESCC, transcriptional reprogramming during tumor progression and metastasis involves gain or loss of several thousand enhancers and alterations in hundreds of putative SEs 55. Transcriptional reprogramming mediated by SEs also regulates CRC development and progression. Inhibition of *KDM6* induces global enhancer reprogramming, especially in the SE-associated genes such as *ID1* that control stemness of CRC cells 56. Liver metastasis in CRC patients is also induced by transcriptional reprogramming. Liver-specific TFs such as FOXA2 and HNF1A bind to altered TEs and SEs in the CRC cells and activate liver-specific gene transcription that drives liver metastasis 57. Furthermore, HNF1A is overexpressed and HNF1A-binding motifs are enriched in the SEs during liver metastasis in CRC 58. Transcriptional reprogramming mediated by SEs also promotes HCC progression. *SIRT7* SE is an acquired SE in nonalcoholic fatty liver disease-related HCC that drives metabolic reprogramming and promotes oncogenic activity through SIRT7-mediated chromatin deregulation of tumor-suppressor genes 59. Xiao et al. performed single-cell holo-transcriptome sequencing and demonstrated genome-wide SE remodeling during the malignant transformation of HCC cells based on data from the 60. In another study, micro-scale chromatin profiling of primary GC tissues showed genome-wide reprogramming of the SE landscape and dense co-occupancy of transcription factors CDX2 and HNF4α, which promote aberrant expression of oncogenes 61. These data demonstrate that understand the mechanisms of SE-mediated transcription and reprogramming of oncogene transcription provide more therapeutic strategies for targeting digestive tumors.

### SE-driven oncogenes promote proliferation and metastasis of cancer cells

SEs are potential targets for inhibiting the expression levels of SE-related oncogenes to reduce tumor cell proliferation and migration. In a subset of esophageal squamous cell carcinoma (ESCC), interactions between the bromodomain protein BRDT and the △Np63 transcription factor in the SE regions of squamous phenotype-related genes such as *KRT14*, *FAT2*, and *PTHLH* increase their transcription levels, thereby promoting ESCC cell migration 62. Therefore, preferential degradation of BRDT by MZ1 reverses the activation of these oncogenes 62. Se-driven AJUBA is activated by TCF4 and regulates migration and invasiveness of HCC cells 63.

Differential H3K27Ac marks were identified in the enhancer regions of SE-driven oncogenes such as *c-MYC*,* MED1*, *OCT4*, *NANOG*, and *SOX2* in the PDAC cell lines 64. However, treatment with GZ17-6.02, a cocktail of natural anticancer agents, reduced progression of PDAC cells by decreasing the H3K27ac levels in the hyperacetylated SE regions of these oncogenes 64. Furthermore, triptolide, a bioactive compound extracted from the Chinese herb *Tripterygium wilfordii*, inhibits proliferation and migration of PDAC cells by disrupting SEs and downregulating the expression levels of SE-driven genes such as* BRD4*, *MYC*, and* RNA pol Ⅱ* 65.

### Tumor diagnostic and prognostic biomarkers

In general, high expression levels of SE-related oncogenes are associated with poor prognosis of cancer patients 66. Huang et al.reported that a prognostic signature of six enhancer-associated differentially expressed genes (DEGs) showed superior performance in predicting the survival outcomes of HCC patients 67. The co-operative interaction between DHX37 and PLRG1 in the promoter and SE regions induces transcription of *cyclin D1*, and promotes proliferation of HCC cells; high cyclin D1 expression is associated with poor prognosis of HCC patients 68. These data demonstrate that SE-induced expression of oncogenes and tumor suppressor genes is associated with the diagnosis and prognosis of digestive system tumors and may contribute to the development of precision medicine.

### Regulation of immune evasion

Immune evasion is an important mechanism that facilitates tumor cell survival, growth, and metastasis. Recent studies have shown the relationship between SEs and the tumor immune escape mechanisms. The immune checkpoint blockade proteins, programmed cell-death protein 1 (PD-1) and programmed cell-death 1 ligand 1 (PD-L1), play an important role in determining the tumor immune microenvironment and anti-cancer immune response 69. The synchronous expression of PD-L1 and PD-L2 is driven by the PD-L1L2-SE in the tumor cells; inhibition of PD-L1L2-SE significantly reduces the expression levels of PD-L1 and PD-L2 and increases the lethality of the cytotoxic T cells 70. CD47 is expressed on the surface of macrophages and other immune cells and inhibits phagocytosis by binding to the SIRPa receptor 71. In breast cancer, CD47 expression is upregulated by a CD47-associated SE 72. The upregulation of SE-regulated IL-20RA is associated with increased immune evasion of CRC cells through oncogenic and immune response pathways that reduce infiltration of N1 neutrophils, M1 macrophages, and T cells 25.

### Noncoding RNAs associated with SEs in digestive system tumors

In addition to regulating the expression of protein-coding genes to directly regulate biological processes, SEs indirectly regulate the biological functions of cancer cells by activating the expression of ncRNAs such as miRNAs, eRNAs, and lncRNAs 31. In the following, we discuss the roles and mechanisms of these SE-associated ncRNAs in tumor progression.

#### MicroRNAs

SEs modulate tumor malignancy by regulating the tissue-specific expression levels of various miRNAs. For example, the SE of KLF6 regulates the proliferation of HCC cells by upregulating p21 and p53, whereas deletion of KLF6 SE inhibits HCC proliferation via miR-1301 overexpression 73. MiR-122 is another SE-driven liver-specific miRNA that is highly expressed in the newborn and adult liver tissues. MiR-122 participates in HCC growth and progression by directly targeting the regulators of Hippo signaling pathway 74.

#### Enhancer RNAs

Enhancer RNAs (eRNAs) are a subfamily of noncoding RNAs transcribed in the enhancer region 75-80. They regulate transcription of specific genes by generating and stabilizing SE-gene promoter loops 37. The overexpression of HPSE, an SE-derived eRNA, promotes GC progression by regulating the hnRNPU/p300/EGR1/HPSE axis 81. Furthermore, the overexpression of UCA1 eRNA in GC increases the AMOT-YAP interactions and activates the Hippo-YAP signaling pathway 82, 83. YAP activation induces apoptosis of the GC cells by increasing Bax protein levels and decreasing Bcl-2 protein levels 84.

#### LncRNAs

LncRNAs are noncoding RNAs that are longer than 200 nucleotides. The dysregulation of lncRNAs is associated with tumorigenesis. SE-associated lncRNAs such as HOTAIR, XIST, SNHG5, and LINC000940 regulate the expression of ESCC hallmark genes 85. The binding of TCF3 and KLF5 transcription factors to the SE region induces the expression of LINC00094, which promotes growth and survival of ESCC cells 85. TP63 transcription factor induces SE-dependent transcription of LINC01503, which subsequently promotes proliferation, migration, and invasion of squamous cell carcinoma cells via the MAPK and Akt signaling pathways 86. In ESCC cells, lncRNA CCAT1 is upregulated through the binding of master transcription factors such as TP63, SOX2, and KLF5 to the SE region; CCAT1 overexpression drives ESCC proliferation and progression 86-88.

SE-associated lncRNAs also regulate CRC growth and progression by modulating the expression level of several CRC-related genes 89. The overexpression of SE-regulated lncRNA RP11-569A11.1 inhibits CRC malignancy by increasing IFIT2 expression levels 89. Conversely, the overexpression of SE-regulated lncRNA AC005592.2 promotes CRC progression 90. In CRC tissues and cells, *MYC* transcription is regulated via long-range chromatin looping induced by the SE-regulated lncRNA CCAT1-L, which is transcribed 515 kb upstream of the *MYC* promoter 35. Furthermore, transcription levels of dysregulated CRC-related lncRNAs are directly regulated by the 5-hydroxymethylcytosine (5hmC) levels in the genomic loci or through aberrant activities of 5hmC -modified TEs, SEs and promoters 91.

LncRNAs are novel candidate oncogenes and potential diagnostic biomarkers for HCC. The overexpression of SE-regulated lncRNAs HCCL5 promotes survival, migration, and EMT of HCC cells, and correlates with poor survival outcomes of HCC patients 38. LncRNA-DAW promotes HCC cell proliferation by activating the Wnt/β-catenin pathway 92. Overall, the above findings suggest that SEs and SE-regulated ncRNAs play a critical role in the progression of digestive system tumors through epigenetic regulation and modulation of oncogenic signaling pathways.

## Targeting super-enhancers as a therapeutic strategy in digestive system tumors

**Figure 3:**
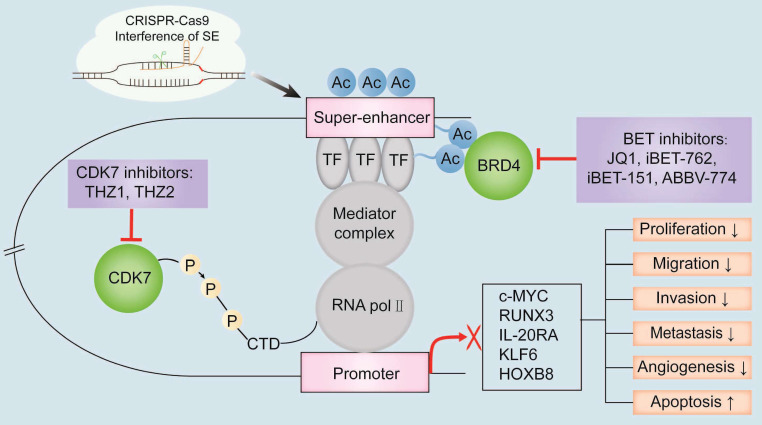
**The known oncogenic SE-targeting small molecule inhibitors and gene-editing mechanisms.** BET inhibitors such as JQ1, iBET-762, iBET-151, and ABBV-774 suppress transcription of oncogenes by either binding to the BET bromodomains or displacing the BET bromodomains. Thus, the interactions between BRD4 and the acetylated sites in the SE region are blocked. CDK7 inhibitors such as THZ1 and THZ2 inhibit the transcription of SE-associated oncogenes by binding covalently to CDK7 and reduce the phosphorylation of the RNA pol II C-terminal domain. CRISPR-Cas9 gene-editing system modulates SE-induced oncogene transcription by remodeling the epigenetic landscapes at the sgRNA-targeted enhancers and associated genes. Gene editing is a convenient tool to analyze the functions of SEs and discover new targets for cancer treatment. Note: TF, transcription factor; H3K27ac, acetylation of histone 3 lysine 27; P, phosphate group; RNA pol II, RNA polymerase II.

Specific inhibition of SEs formation and SE-dependent activation of oncogene transcription represents a novel strategy for cancer therapy. Inhibitors targeting SE components bromodomain and extra terminal domain (BET) or cyclin-dependent kinase (CDK), have shown immense potential in cancer therapy and are currently undergoing clinical trials to determine the efficacy and safety in treating patients with digestive system tumors (**Table 2**). Furthermore, disruption of the formation and structure of the oncogenic SEs by gene editing technology has emerged as a new potential novel strategy to treat cancers (**Figure 3**).

### Small-molecule inhibitors targeting super-enhancer-related proteins

#### BET inhibitors

The BET protein family members such as BRD2, BRD3, BRD4, and BRDT are important regulators of epigenetic modifications and gene transcription 93. Among these, BRD4 occupies the SE regions and regulates gene transcription by binding to H3K27ac 94. BRD4 inhibition disrupts the communication between SEs and their target promoters, and significantly decreases the expression of SE-driven oncogenes, which are essential for cancer progression 95.

Currently, the clinical efficacy of BET inhibitors is being evaluated for various cancers. JQ1 is the most widely studied small-molecule BET inhibitor that blocks SE-induced oncogene transcription 96. JQ1 inhibits proliferation of CRC cells by disrupting Cmyc-SE and downregulating *c-MYC* expression 97. The cell cycle progression and survival of BRAF^V600E^-mutant CRC cells was reduced by treatment with a combination of JQ1 and vemurafenib, a selective inhibitor of mutated BRAF 98. KDM6A-deficient pancreatic cancer cells show aberrant activation of SEs that regulate oncogenes such as *△Np63*, *MYC*, and *RUNX3*, but are but are highly sensitive to BET inhibitors 99.

The clinical efficacy and safety profiles of BET inhibitors including I-BET762 and I-BET151 are currently being evaluated in patients with digestive system tumors. I-BET762 inhibits PDAC cell proliferation and progression by inducing G0/G1 phase cell cycle arrest and cell death 100. I-BET151 inhibits the growth of CRC cells by downregulating the expression of SE-driven IL-20RA 25. However, some clinical trials have reported adverse effects of BET inhibitors, including moderate gastrointestinal toxicity and fatigue, thrombocytopenia, and cardiotoxicity 101-103. Androgen receptor (AR)-dependent transcription in prostate cancer cells is suppressed by the second-generation BET inhibitor ABBV-744, which selectively inhibits the BD2 domain of BET family proteins and displaces BRD4 from SEs with AR 104. The adverse effects of traditional BET inhibitors can be overcome by proteolysis-targeting chimeras, which effectively inhibit solid tumors with low cytotoxicity by degrading the BET proteins 105.

Several solid tumors demonstrate resistance against BRD4 inhibitors. Wang et al. demonstrated that BRD4 phosphorylation at tyrosine 97/98 induced resistance against the BET inhibitors in CRC Cells 106. Furthermore, because methylation status of the promoter region regulates gene expression, drugs targeting epigenetic modifications are also being evaluated for cancer therapy 107.

#### CDK inhibitors

CDKs regulate cell cycle and transcription by controlling the initiation and elongation of RNA pol Ⅱ 108, 109. Selective inhibition of CDK7 significantly reduces transcription of oncogenic TFs, especially those associated with SEs 110. Therefore, inhibition of CDK7 is a promising therapeutic strategy against digestive system tumors.

THZ1 is a covalent CDK7 inhibitor that inhibits RNA pol Ⅱ C-terminal domain phosphorylation 108. This decreases the number of RNA pol Ⅱ molecules in the SE region and inhibits the transcription of SE-related oncogenes 94. Low-dose THZ1 significantly reduces SE-driven transcription of *PAK4, RUNX1, DNAJB1, SREBF2*, and *YAP1* oncogenes in the ESCC cells 111. THZ1 also suppresses the growth of ESCC cells by downregulating the expression of lncRNA LINC00094 85. Huang et al. 112 showed that nanoparticles with high drug loading of JQ1 and THZ1 (J/T@8P4s) effectively suppressed SE-associated oncogenic transcription in the PDAC cells. In the *in vitro* and *in vivo* HCC models, THZ1 exerts significant antitumor effects by selectively suppressing the expression levels of SE-associated oncogenes 113. SY-1365 is a highly potent and selective CDK7 inhibitor that inhibits the growth of various cancers in both *in vivo* and *in vitro* experimental models and is currently undergoing clinical trials 110. CDK7/9 inhibitor SNS-032 significantly inhibits the growth of ESCC cells and xenografts 114. Therefore, inhibition of CDK7 and CDK9 is a promising therapeutic strategy for digestive system tumors.

### Targeting oncogenic SEs by gene editing

In recent years, gene-editing technology has emerged as a convenient tool to study the functions of genes by correcting the mutated genes or modifying the DNa sequences of genes in human diseases. The CRISPR-Cas9 system is the most efficient gene-editing tool that is used for basic and clinical research. Gene-editing technology is also used to design therapeutics targeting oncogenic or disease-related SEs.

CRISPR-Cas9 technology can be used to prevent the formation of disease-related SEs which are generated by genetic mutations such as base insertions, deletions, or substitutions and chromatin rearrangements. It has also been used to investigate mechanisms related to tumorgenesis and identify new drug targets 115. CRISPR-Cas9 technology can be used to study the cooperation between individual enhancers within the SE region by knocking out individual enhancers 116. Moreover, the CRISPR-Cas9 system can also alter the functions of SEs by deleting specific DNA sequences and promoting nonhomologous recombinant repair 117. For example, disruption of the SE regions in the RUNX1 gene by the CRISPR-Cas9 system induces apoptosis of leukemic cells 118. Moreover, the combination of advanced genome-wide sequencing and CRISPR-Cas9 technology is used to investigate the *in vivo* functions of SEs in the mammary gland genome 119. This strategy may provide new insights into investigating SEs-associated tumorigenesis in the future.

CRISPR-Cas9 technology has also been used to study the function of SE components in the digestive system tumors. Deletion of the *TP63* promoter or individual components of the *TP63* SE through CRISPR-Cas9 decreases the expression levels of *TP63* and inhibits the proliferation of the ESCC cells, thereby indicating the association of *TP63* with tumor malignancy 87. Furthermore, CRISPR-Cas9-mediated knockdown of the SE complex components such as CDK7, BRD4, EP300, and MED1 significantly inhibited the proliferation of HCC cells 113. The CRISPR-Cas9 system can also be used to alter SE-driven oncogene expression through single nucleotide polymorphisms (SNPs), which alter the gene expression patterns by disrupting the formation and interaction of the SEs 120. H3K4me1 and H3K27ac markers are enriched in the SE region of SNP rs6854845, which is a risk factor associated with colon cancer 121. Genome editing by CRISPR-Cas9 alters the transcription of genes by modulating the interactions between SE-associated genes and SNPs 121.

Although CRISPR-Cas9 is a highly efficient and convenient tool, it has drawbacks. In some cases, ineffective base editing and off-target effects limit the application of CRISPR-Cas9 technology 122. Therefore, the efficacy and safety of gene-editing technology in targeting disease-related SEs requires careful characterization for clinical applications. Li et al. developed a more efficient enCRISPRa system for enhancer activation and the enCRISPRi system for enhancer inhibition to analyze the *in situ* and *in vivo* functions of enhancers 123. Liu et al. used the CRISPR affinity purification *in situ* of regulatory elements (CAPTURE) proteomics approach to identify locus-specific chromatin-regulating protein complexes and long-range DNA interactions without bias 124. This proteomics approach captures protein complexes that bind to SE with greater sensitivity and specificity and can be used to find new targets to develop small‑molecule inhibitors for cancer therapy. A new precise gene-editing tool, Prime Editor, has been developed to edit SE formation and overcome the non-specific base editing and off‑target effects of the CRISPR-Cas9 system 125. Overall, there is great scope for the application of gene-editing tools in SE editing for cancer therapy.

## Conclusion

The aberrant activity of SEs drives oncogene transcription in digestive system tumors and promotes tumor cell proliferation, migration, and malignancy. SE-associated miRNAs, eRNAs, and lncRNAs also promote growth and progression of digestive system tumors indirectly. SEs are cell-type-specific. Hence, they are potential biomarkers for identifying and classifying different subtypes of digestive system tumors. However, further studies are needed to determine frequently dysregulated SEs and the downstream oncogenic signaling pathways in different digestive system tumors.

SEs are promising therapeutic targets in digestive system tumors. However, the potential target of gene editing at the DNA level is still unclear because SEs occupy large regions of DNA. At present, SE-targeted therapy is focused on small-molecule inhibitors of BET and CDKs, which have shown significant antitumor efficacy in several pre-clinical studies and are currently undergoing clinical trials (**Table 2**). However, the clinical application of BET and CDKs inhibitors are limited because they interfere with the transcription of many proteins and cause significant toxicity and adverse events. SY-1365 is a selective CDK7 inhibitor that entered the phase I clinical trial in 2017 126. However, preliminary findings demonstrate that the efficacy of SY-1365 is average. Therefore, effective CDK7-targeted anti-cancer therapyrequires further verification in the future. Moreover, further investigations are necessary to determine the efficacy and safety profiles of SE-targeting inhibitors in combination with other antitumor agents such as chemotherapy drugs and anti-tumor monoclonal antibody drugs in advanced or drug-resistant digestive system tumors.

Several studies have demonstrated that CRISPR-Cas9 gene-editing technology can be used to abolish SE formation in cancer cells. This modern technology shows great promise for individualized treatment of cancer patients based on the status of many SEs. In conclusion, SEs play important roles in digestive system tumors by driving oncogenic expression and oncogenic signaling pathways. Further studies are necessary to unravel the complexity of SE-related mechanisms, their clinical significance as therapeutic targets, and investigate more effective therapies targeting SE.

## Figures and Tables

**Table 1:** SE-related genes in the digestive system tumors.

Tumor process	SE-related genes	Mechanisms	Drugs/ Inhibitors	Tumor type	Reference
Migration	PHF19 and TBC1D16	SEs bound by multiple TFs		CRC	45
Proliferation	ELF3, KLF5, GATA6, and EHF	Formation of interconnected transcriptional circuit		EAC	46
Invasiveness	HOXB8	MYC-regulated SE and BACH1	JQ1, iBET-762	CRC	48
	IGF2	de novo SE formation		CRC	49
Aggressive phenotype	EVI1	Configuration of the active enhancer chromatin		PDAC	50
Migration and EMT	TGFBR2	TGF-β signaling	JQ1	PDAC	51
Progression	ZFP36L2	Tandem duplication (TD) of the ZFP36L2 SE		GC	53
Proliferation	IL-6		JQ-1 and I-BET-762	PADC	54
Migration	KRT14, FAT2, and PTHLH	Interaction with ΔNp63 and BRDT	MZ1	ESCC	62
Invasion and metastasis	AJUBA	Akt/GSK-3β/Snail signaling pathway and TCF4 binding		HCC	63
Progression	c-MYC, MED1, OCT-4, NANOG, and SOX2	Differential H3K27ac of the master transcription factor genes	GZ17-6.02	PDAC	64
	BRD4, MYC, RNA Pol II, and Collagen 1	Reprogramming of cellular crosstalk and signaling	Triptolide	PDAC	65
	CDX2 and HNF4α	Dense co-occupancy of transcription factors		GC	61
Recurrence	ID1	Global enhancer reprogramming	GSK-J4	CRC	56
Metastasis	FOXA2 and HNF1A	Directional transcription and reprogramming		CRC	57, 58
Progression	Sirtuin 7	Epigenomic reprogramming and SIRT7-mediated chromatin deregulation		HCC	59
Proliferation and migration	SPHK1	SE landscape reprogramming	CBP30, JQ1, and THZ1	HCC	113
Proliferation/metastasis and immune evasion	IL-20RA	Oncogenic and immune response pathways	JQ-1, iBET-151	CRC	25
Proliferation	KLF6	MiR-1301 and a p53-dependent manner		HCC	73
Progression	HPSE	hnRNPU/p300/EGR1/HPSE axis		GC	81
Apoptosis	UCA1	AMOTp130-YAP and Hippo-YAP signaling		GC	83
Cell growth and survival	LINC00094	Binding of TCF3 and KLF5 in the SE regions		ESCC	85
Proliferation, migration, and invasion	LINC01503	Binding of TP63 in the SE regions		ESCC	86
Proliferation	TP63, SOX2, and KLF5	The interconnected transcriptional network	ARV-771	ESCC	87
Progression, metastasis	RP11-569A11.1	Positive regulation of IFIT2 expression		CRC	89
Progression	AC005592.2	Positive regulation of OLFM4 expression		CRC	90
	CCAT1-L	MYC transcriptional regulation and long-range chromatin looping	ASO	CRC	35
Migration, and EMT	HCCL5	Interaction of ZEB1 at both SE and promoter regions		HCC	38
Proliferation	lncRNA-DAW	Wnt/β-catenin pathway		HCC	92
Metastasis	ΔNp63, MYC, and RUNX3	Loss of KDM6A	JQ1, iBET-151	PDAC	99
Proliferation	PAK4, RUNX1, DNAJB1, SREBF2, and YAP1	RAF/MEK/ERK and PI3K/AKT pathways	THZ1, KPT-9274	ESCC	111
Proliferation	ZFP36L2	DNA hypermethylation			127
Proliferation, migration, apoptosis	RAI14	Akt signaling pathway		GC	128
Proliferation	c-MYC	Hyperactive Wnt/β-catenin/TCF signaling	JQ1, iBET-151	CRC	97
Proliferation	FOXQ1	Significant enrichment of hypomethylated SE loci		CRC	129
Proliferation	CD9 and PLEKHG6	Long-distance interactions of super-enhancer SNP rs11064124 and the corresponding target genes		CRC	130
Growth and progression	ME3	SE-mediated constitutive activation system		HCC	131
	C/EBPβ	Aberrant enhancer hypomethylation and transcriptional reprogramming		HCC	132
EMT and metastasis	QKI	Regulation of the YY1/p65/p300 complex		HCC	133
Autophagy	ZNF263	Endoplasmic reticulum stress		HCC	134
Metastasis and drug resistance	Tex10	Positive regulation of embryonic stem cell super-enhancers and the STAT3 signaling pathway		HCC	135
	GATA6, FOS, FOXP1, FOXP4, KLF4, LF3, NFIX, CUX1, and SSBP3	Regulation other fate-determining TFs		PDAC	136
	Kras^G12D^	Chromatin remodeling across genomic areas		PDAC	137
	MYC	High amplification and core pancreas-specific SE regions		PDAC	138
Tumorigenesis	MYC or Kras	Epigenetic configuration of latent pancreas regenerative super-enhancer program	JQ1	PDAC	139
Proliferation	ΔNp63	Interdependent transcription factor network		PDAC	140

ESCC, Esophageal squamous cell carcinoma; EAC, Esophageal adenocarcinoma; GC, Gastric cancer; CRC, cancer; HCC, Hepatocellular carcinoma; PDAC, Pancreatic ductal adenocarcinoma; SNP, Single nucleotide polymorphism

**Table 2:** SE-related inhibitors for the potential treatment of digestive system tumors.

Target	Compound	Status	Identifier	Tumor type
BET/ BRD4	SYHA1801	Phase 1, recruiting	NCT04309968	Advanced solid tumors
	SF1126	Phase 1, active, not recruiting	NCT03059147	HCC
	BMS-986158, BMS-986378	Phase 1, recruiting	NCT03936465	Solid Tumor, Childhood
	INCB057643	Phase 1/2, terminated	NCT02959437	CRC
	INCB054329	Phase 1/2, terminated	NCT02431260	CRC / PAAD
	GSK525762	Phase 1, completed	NCT01587703	CRC
	INCB057643	Phase 1/2, terminated	NCT02711137	CRC
	Birabresib (MK-8628)	Phase 1, completed	NCT02259114	PDAC
	INCB057643	Phase 1/2, terminated	NCT02711137	PDAC
	AZD5153	Phase 1, completed	NCT03205176	PDAC, malignant solid tumors
	ZEN-3694	Phase 1, recruiting	NCT04840589	Metastatic / recurrent malignant solid neoplasm
CDK7	SY-5609	Phase 1, recruiting	NCT04247126	PDAC
	SY-1365	Phase 1, terminated	NCT03134638	Advanced solid tumors
	CT7001	Phase 1/2, active, not recruiting	NCT03363893	Advanced solid malignancies
CDK9	TP-1287	Phase 1, recruiting	NCT03604783	Advanced solid tumors
	Fadraciclib (CYC065)	Phase 1/2, recruiting	NCT04983810	Solid tumor
	KB-0742	Phase 1, recruiting	NCT04718675	Relapsed / refractory solid tumors

BET, Bromodomain and extra terminal; BRD4, Bromodomain containing protein 4; CDK, Cyclin-dependent kinase; HCC, Hepatocellular Carcinoma; CRC, Colorectal cancer; PAAD, Pancreatic adenocarcinoma; PDAC, Pancreatic ductal adenocarcinoma.

## Abbreviations

SEs: super-enhancers
TFs: transcription factors
RNA pol II: RNA polymerase II
H3K4me1: histone H3 lysine4 methylation
H3K27ac: histone H3 lysine27 acetylation
ChIP-seq: chromatin immunoprecipitation sequencing
ESCs: embryonic stem cells
TEs: typical enhancers
CDK7: cyclin-dependent kinase 7
BRD4: bromodomain protein 4
ncRNAs: non-coding RNAs
miRNAs: microRNAs
lncRNAs: long non-coding RNAs
circRNAs: circular RNAs
eRNAs: enhancer RNAs
LLPS: liquid-liquid phase separation
IDRs: intrinsically disordered regions
EBV: Epstein-Barr virus
HPV: Human papillomavirus
HTLV-I: human T-lymphotropic virus type Ⅰ
HBV: Hepatitis B virus
EAC: esophageal adenocarcinoma
CRC: colorectal cancer
PDAC: pancreatic ductal adenocarcinoma
GC: gastric cancer
ESCC: esophageal squamous cell carcinoma
HCC: hepatocellular carcinoma
EMT: epithelial-mesenchymal transition
BET: bromodomain and extra terminal domain
CDKs: cyclin-dependent kinases
SNPs: single nucleotide polymorphisms

## References

[1] Nagaraju GP, Kasa P, Dariya B, Surepalli N, Peela S, Ahmad S (2021). Epigenetics and therapeutic targets in gastrointestinal malignancies. Drug Discov Today.

[2] Jin N, George TL, Otterson GA, Verschraegen C, Wen H, Carbone D (2021). Advances in epigenetic therapeutics with focus on solid tumors. Clinical epigenetics.

[3] Arrowsmith CH, Bountra C, Fish PV, Lee K, Schapira M (2012). Epigenetic protein families: a new frontier for drug discovery. Nat Rev Drug Discov.

[4] Vedeld HM, Goel A, Lind GE (2018). Epigenetic biomarkers in gastrointestinal cancers: The current state and clinical perspectives. Seminars in cancer biology.

[5] Whyte WA, Orlando DA, Hnisz D, Abraham BJ, Lin CY, Kagey MH (2013). Master transcription factors and mediator establish super-enhancers at key cell identity genes. Cell.

[6] Li GH, Qu Q, Qi TT, Teng XQ, Zhu HH, Wang JJ (2021). Super-enhancers: a new frontier for epigenetic modifiers in cancer chemoresistance. J Exp Clin Cancer Res.

[7] Hnisz D, Abraham BJ, Lee TI, Lau A, Saint-Andre V, Sigova AA (2013). Super-enhancers in the control of cell identity and disease. Cell.

[8] Qu J, Ouyang Z, Wu W, Li G, Wang J, Lu Q (2020). Functions and Clinical Significance of Super-Enhancers in Bone-Related Diseases. Frontiers in cell and developmental biology.

[9] Zabidi MA, Stark A (2016). Regulatory Enhancer-Core-Promoter Communication via Transcription Factors and Cofactors. Trends in genetics: TIG.

[10] Sur I, Taipale J (2016). The role of enhancers in cancer. Nature reviews Cancer.

[11] Kundaje A, Meuleman W, Ernst J, Bilenky M, Yen A, Heravi-Moussavi A (2015). Integrative analysis of 111 reference human epigenomes. Nature.

[12] He Y, Long W, Liu Q (2019). Targeting Super-Enhancers as a Therapeutic Strategy for Cancer Treatment. Front Pharmacol.

[13] Gryder BE, Wu L, Woldemichael GM, Pomella S, Quinn TR, Park PMC Chemical genomics reveals histone deacetylases are required for core regulatory transcription. 2019; 10: 3004.

[14] Hnisz D, Schuijers J, Lin CY, Weintraub AS, Abraham BJ, Lee TI (2015). Convergence of developmental and oncogenic signaling pathways at transcriptional super-enhancers. Molecular cell.

[15] Mansour MR, Abraham BJ, Anders L, Berezovskaya A, Gutierrez A, Durbin AD (2014). Oncogene regulation. An oncogenic super-enhancer formed through somatic mutation of a noncoding intergenic element. Science (New York, NY).

[16] Ing-Simmons E, Seitan VC, Faure AJ, Flicek P, Carroll T, Dekker J (2015). Spatial enhancer clustering and regulation of enhancer-proximal genes by cohesin. Genome research.

[17] Sabari BR, Dall'Agnese A, Boija A, Klein IA, Coffey EL, Shrinivas K (2018). Coactivator condensation at super-enhancers links phase separation and gene control. Science (New York, NY).

[18] Visel A, Blow MJ, Li Z, Zhang T, Akiyama JA, Holt A (2009). ChIP-seq accurately predicts tissue-specific activity of enhancers. Nature.

[19] Jin F, Li Y, Dixon JR, Selvaraj S, Ye Z, Lee AY (2013). A high-resolution map of the three-dimensional chromatin interactome in human cells. Nature.

[20] Boyle AP, Davis S, Shulha HP, Meltzer P, Margulies EH, Weng Z (2008). High-resolution mapping and characterization of open chromatin across the genome. Cell.

[21] Ohkura N, Yasumizu Y, Kitagawa Y, Tanaka A, Nakamura Y, Motooka D (2020). Regulatory T Cell-Specific Epigenomic Region Variants Are a Key Determinant of Susceptibility to Common Autoimmune Diseases. Immunity.

[22] Sun X, Ren Z, Cun Y, Zhao C, Huang X, Zhou J (2020). Hippo-YAP signaling controls lineage differentiation of mouse embryonic stem cells through modulating the formation of super-enhancers. Nucleic acids research.

[23] Lu B, He Y, He J, Wang L, Liu Z, Yang J (2020). Epigenetic Profiling Identifies LIF as a Super-enhancer-Controlled Regulator of Stem Cell-like Properties in Osteosarcoma. Mol Cancer Res.

[24] Binnewies M, Roberts EW, Kersten K, Chan V, Fearon DF, Merad M (2018). Understanding the tumor immune microenvironment (TIME) for effective therapy. Nat Med.

[25] Yu D, Yang X, Lin J, Cao Z, Lu C, Yang Z (2021). Super-Enhancer Induced IL-20RA Promotes Proliferation/Metastasis and Immune Evasion in Colorectal Cancer. Frontiers in oncology.

[26] Natsume A, Hirano M, Ranjit M, Aoki K, Wakabayashi T (2019). Aberrant Transcriptional Regulation of Super-enhancers by RET Finger Protein-histone Deacetylase 1 Complex in Glioblastoma: Chemoresistance to Temozolomide. Neurol Med Chir (Tokyo).

[27] Shang S, Yang J, Jazaeri AA Chemotherapy-Induced Distal Enhancers Drive Transcriptional Programs to Maintain the Chemoresistant State in Ovarian Cancer. 2019; 79: 4599-611.

[28] Saint-André V, Federation AJ, Lin CY, Abraham BJ, Reddy J, Lee TI (2016). Models of human core transcriptional regulatory circuitries. Genome research.

[29] Jiang Y, Jiang YY, Lin DC (2021). Super-enhancer-mediated core regulatory circuitry in human cancer. Computational and structural biotechnology journal.

[30] Zhang T, Song X, Zhang Z, Mao Q, Xia W, Xu L Aberrant super-enhancer landscape reveals core transcriptional regulatory circuitry in lung adenocarcinoma. 2020; 9: 92.

[31] Wang Y, Nie H, He X, Liao Z, Zhou Y, Zhou J (2020). The emerging role of super enhancer-derived noncoding RNAs in human cancer. Theranostics.

[32] Suzuki HI, Young RA, Sharp PA (2017). Super-Enhancer-Mediated RNA Processing Revealed by Integrative MicroRNA Network Analysis. Cell.

[33] Pefanis E, Wang J, Rothschild G, Lim J, Kazadi D, Sun J (2015). RNA exosome-regulated long non-coding RNA transcription controls super-enhancer activity. Cell.

[34] Huang S, Li X, Zheng H, Si X, Li B, Wei G (2019). Loss of Super-Enhancer-Regulated circRNA Nfix Induces Cardiac Regeneration After Myocardial Infarction in Adult Mice. Circulation.

[35] Xiang JF, Yin QF, Chen T, Zhang Y, Zhang XO, Wu Z (2014). Human colorectal cancer-specific CCAT1-L lncRNA regulates long-range chromatin interactions at the MYC locus. Cell research.

[36] Mao R, Wu Y, Ming Y, Xu Y, Wang S, Chen X (2019). Enhancer RNAs: a missing regulatory layer in gene transcription. Science China Life sciences.

[37] Tan Y, Li Y, Tang F (2020). Oncogenic seRNA functional activation: a novel mechanism of tumorigenesis. Mol Cancer.

[38] Peng L, Jiang B, Yuan X, Qiu Y, Peng J, Huang Y (2019). Super-Enhancer-Associated Long Noncoding RNA HCCL5 Is Activated by ZEB1 and Promotes the Malignancy of Hepatocellular Carcinoma. Cancer Res.

[39] Wu M, Shen J (2019). From Super-Enhancer Non-coding RNA to Immune Checkpoint: Frameworks to Functions. Frontiers in oncology.

[40] Hnisz D, Shrinivas K, Young RA, Chakraborty AK, Sharp PA (2017). A Phase Separation Model for Transcriptional Control. Cell.

[41] Zhang J, Yue W, Zhou Y, Liao M, Chen X, Hua J (2021). Super enhancers-Functional cores under the 3D genome. Cell proliferation.

[42] Hyman AA, Weber CA, Jülicher F (2014). Liquid-liquid phase separation in biology. Annual review of cell and developmental biology.

[43] Sabari BR, Dall'Agnese A Coactivator condensation at super-enhancers links phase separation and gene control. 2018; 361.

[44] Ahn JH, Davis ES (2021). Phase separation drives aberrant chromatin looping and cancer development. Nature.

[45] Li QL, Lin X, Yu YL, Chen L, Hu QX, Chen M (2021). Genome-wide profiling in colorectal cancer identifies PHF19 and TBC1D16 as oncogenic super enhancers. Nature communications.

[46] Chen L, Huang M, Plummer J, Pan J, Jiang YY, Yang Q (2020). Master transcription factors form interconnected circuitry and orchestrate transcriptional networks in oesophageal adenocarcinoma. Gut.

[47] Ma S, Zhou B, Yang Q, Pan Y, Yang W, Freedland SJ (2021). A Transcriptional Regulatory Loop of Master Regulator Transcription Factors, PPARG, and Fatty Acid Synthesis Promotes Esophageal Adenocarcinoma. Cancer research.

[48] Ying Y, Wang Y, Huang X, Sun Y, Zhang J, Li M (2020). Oncogenic HOXB8 is driven by MYC-regulated super-enhancer and potentiates colorectal cancer invasiveness via BACH1. Oncogene.

[49] Weischenfeldt J, Dubash T, Drainas AP, Mardin BR, Chen Y, Stütz AM (2017). Pan-cancer analysis of somatic copy-number alterations implicates IRS4 and IGF2 in enhancer hijacking. Nat Genet.

[50] Kim HR, Yim J, Yoo HB, Lee SE, Oh S, Jung S (2021). EVI1 activates tumor-promoting transcriptional enhancers in pancreatic cancer. NAR cancer.

[51] Zhu X, Zhang T, Zhang Y, Chen H, Shen J, Jin X (2020). A super-enhancer controls TGF- β signaling in pancreatic cancer through downregulation of TGFBR2. Cell Signal.

[52] Jones S, Zhang X, Parsons DW, Lin JC, Leary RJ, Angenendt P (2008). Core signaling pathways in human pancreatic cancers revealed by global genomic analyses. Science (New York, NY).

[53] Xing R, Zhou Y, Yu J, Yu Y, Nie Y, Luo W (2019). Whole-genome sequencing reveals novel tandem-duplication hotspots and a prognostic mutational signature in gastric cancer. Nature communications.

[54] Bao Y, Wu Y, Tao B, Sun R, Lin T, Zheng Y (2020). Super-enhancers modulate interleukin-6 expression and function in cancers. Translational cancer research.

[55] Ye B, Fan D, Xiong W, Li M, Yuan J, Jiang Q (2021). Oncogenic enhancers drive esophageal squamous cell carcinogenesis and metastasis. Nature communications.

[56] Zhang J, Ying Y, Li M, Wang M, Huang X, Jia M (2020). Targeted inhibition of KDM6 histone demethylases eradicates tumor-initiating cells via enhancer reprogramming in colorectal cancer. Theranostics.

[57] Teng S, Li YE, Yang M, Qi R, Huang Y, Wang Q (2020). Tissue-specific transcription reprogramming promotes liver metastasis of colorectal cancer. Cell research.

[58] Cai C, Bi D, Bick G, Wei Q, Liu H, Lu L (2021). Hepatocyte nuclear factor HNF1A is a potential regulator in shaping the super-enhancer landscape in colorectal cancer liver metastasis. FEBS letters.

[59] Wu F, Xu L, Tu Y, Cheung OK, Szeto LL, Mok MT (2022). Sirtuin 7 super-enhancer drives epigenomic reprogramming in hepatocarcinogenesis. Cancer letters.

[60] Xiao Z, Cheng G, Jiao Y, Pan C, Li R, Jia D (2018). Holo-Seq: single-cell sequencing of holo-transcriptome. Genome biology.

[61] Ooi WF, Xing M, Xu C, Yao X, Ramlee MK, Lim MC (2016). Epigenomic profiling of primary gastric adenocarcinoma reveals super-enhancer heterogeneity. Nature communications.

[62] Wang X, Kutschat AP, Yamada M, Prokakis E, Böttcher P, Tanaka K (2021). Bromodomain protein BRDT directs ΔNp63 function and super-enhancer activity in a subset of esophageal squamous cell carcinomas. Cell death and differentiation.

[63] Zhang C, Wei S, Sun WP, Teng K, Dai MM, Wang FW (2020). Super-enhancer-driven AJUBA is activated by TCF4 and involved in epithelial-mesenchymal transition in the progression of Hepatocellular Carcinoma. Theranostics.

[64] Ghosh C, Paul S, Dandawate P, Gunewardena SS, Subramaniam D, West C (2019). Super-enhancers: novel target for pancreatic ductal adenocarcinoma. Oncotarget.

[65] Noel P, Hussein S, Ng S, Antal CE, Lin W, Rodela E (2020). Triptolide targets super-enhancer networks in pancreatic cancer cells and cancer-associated fibroblasts. Oncogenesis.

[66] Ren X, Zhang L Integrated bioinformatics and experiments reveal the roles and driving forces for HSF1 in colorectal cancer. 2022; 13: 2536-52.

[67] Huang P, Zhang B, Zhao J, Li MD (2022). Integrating the Epigenome and Transcriptome of Hepatocellular Carcinoma to Identify Systematic Enhancer Aberrations and Establish an Aberrant Enhancer-Related Prognostic Signature. Frontiers in cell and developmental biology.

[68] Liu Z, Ye Y RNA helicase DHX37 facilitates liver cancer progression by cooperating with PLRG1 to drive super enhancer-mediated transcription of cyclin D1. 2022.

[69] Goodman A, Patel SP, Kurzrock R (2017). PD-1-PD-L1 immune-checkpoint blockade in B-cell lymphomas. Nat Rev Clin Oncol.

[70] Xu Y, Wu Y, Zhang S, Ma P, Jin X, Wang Z (2019). A Tumor-Specific Super-Enhancer Drives Immune Evasion by Guiding Synchronous Expression of PD-L1 and PD-L2. Cell Rep.

[71] Oldenborg PA (2013). CD47: A Cell Surface Glycoprotein Which Regulates Multiple Functions of Hematopoietic Cells in Health and Disease. ISRN hematology.

[72] Betancur PA, Abraham BJ, Yiu YY, Willingham SB, Khameneh F, Zarnegar M A CD47-associated super-enhancer links pro-inflammatory signalling to CD47 upregulation in breast cancer. 2017; 8: 14802.

[73] Ri K, Kim C, Pak C, Ri P, Om H (2020). The KLF6 Super Enhancer Modulates Cell Proliferation via MiR-1301 in Human Hepatoma Cells. Microrna.

[74] Zhang Y, Tan YY, Chen PP, Xu H Genome-wide identification of microRNA targets reveals positive regulation of the Hippo pathway by miR-122 during liver development. 2021; 12: 1161.

[75] Kim TK, Hemberg M, Gray JM (2015). Enhancer RNAs: a class of long noncoding RNAs synthesized at enhancers. Cold Spring Harbor perspectives in biology.

[76] Beagrie RA, Pombo A (2016). Gene activation by metazoan enhancers: Diverse mechanisms stimulate distinct steps of transcription. BioEssays: news and reviews in molecular, cellular and developmental biology.

[77] Mousavi K, Zare H, Dell'orso S, Grontved L, Gutierrez-Cruz G, Derfoul A (2013). eRNAs promote transcription by establishing chromatin accessibility at defined genomic loci. Molecular cell.

[78] Mousavi K, Zare H, Koulnis M, Sartorelli V (2014). The emerging roles of eRNAs in transcriptional regulatory networks. RNA biology.

[79] Sartorelli V, Lauberth SM (2020). Enhancer RNAs are an important regulatory layer of the epigenome. Nature structural & molecular biology.

[80] Natoli G, Andrau JC (2012). Noncoding transcription at enhancers: general principles and functional models. Annual review of genetics.

[81] Jiao W, Chen Y, Song H, Li D, Mei H, Yang F (2018). HPSE enhancer RNA promotes cancer progression through driving chromatin looping and regulating hnRNPU/p300/EGR1/HPSE axis. Oncogene.

[82] Yao F, Wang Q, Wu Q (2019). The prognostic value and mechanisms of lncRNA UCA1 in human cancer. Cancer management and research.

[83] Lin X, Spindler TJ, de Souza Fonseca MA, Corona RI, Seo JH, Dezem FS (2019). Super-Enhancer-Associated LncRNA UCA1 Interacts Directly with AMOT to Activate YAP Target Genes in Epithelial Ovarian Cancer. iScience.

[84] Ye C, Wang W, Xia G, Yu C, Yi Y, Hua C (2019). A novel curcumin derivative CL-6 exerts antitumor effect in human gastric cancer cells by inducing apoptosis through Hippo-YAP signaling pathway. OncoTargets and therapy.

[85] Wang QY, Peng L, Chen Y, Liao LD, Chen JX, Li M (2020). Characterization of super-enhancer-associated functional lncRNAs acting as ceRNAs in ESCC. Mol Oncol.

[86] Xie JJ, Jiang YY, Jiang Y, Li CQ, Lim MC, An O (2018). Super-Enhancer-Driven Long Non-Coding RNA LINC01503, Regulated by TP63, Is Over-Expressed and Oncogenic in Squamous Cell Carcinoma. Gastroenterology.

[87] Jiang YY, Jiang Y, Li CQ, Zhang Y, Dakle P, Kaur H (2020). TP63, SOX2, and KLF5 Establish a Core Regulatory Circuitry That Controls Epigenetic and Transcription Patterns in Esophageal Squamous Cell Carcinoma Cell Lines. Gastroenterology.

[88] Zhang E, Han L, Yin D, He X, Hong L, Si X (2017). H3K27 acetylation activated-long non-coding RNA CCAT1 affects cell proliferation and migration by regulating SPRY4 and HOXB13 expression in esophageal squamous cell carcinoma. Nucleic acids research.

[89] Chen H, Zheng J, Yan L, Zhou X, Jiang P, Yan F (2021). Super-enhancer-associated long noncoding RNA RP11-569A11.1 inhibited cell progression and metastasis by regulating IFIT2 in colorectal cancer. Journal of clinical laboratory analysis.

[90] Yan L, Chen H, Tang L, Jiang P, Yan F (2021). Super-enhancer-associated long noncoding RNA AC005592.2 promotes tumor progression by regulating OLFM4 in colorectal cancer. BMC Cancer.

[91] Hu H, Shu M, He L, Yu X, Liu X, Lu Y (2017). Epigenomic landscape of 5-hydroxymethylcytosine reveals its transcriptional regulation of lncRNAs in colorectal cancer. British journal of cancer.

[92] Liang W, Shi C, Hong W, Li P, Zhou X, Fu W (2021). Super-enhancer-driven lncRNA-DAW promotes liver cancer cell proliferation through activation of Wnt/β-catenin pathway. Molecular therapy Nucleic acids.

[93] Belkina AC, Denis GV (2012). BET domain co-regulators in obesity, inflammation and cancer. Nature reviews Cancer.

[94] Tang F, Yang Z, Tan Y, Li Y (2020). Super-enhancer function and its application in cancer targeted therapy. NPJ Precis Oncol.

[95] Donati B, Lorenzini E, Ciarrocchi A (2018). BRD4 and Cancer: going beyond transcriptional regulation. Molecular cancer.

[96] Filippakopoulos P, Qi J, Picaud S, Shen Y, Smith WB, Fedorov O (2010). Selective inhibition of BET bromodomains. Nature.

[97] Tögel L, Nightingale R, Chueh AC, Jayachandran A, Tran H, Phesse T (2016). Dual Targeting of Bromodomain and Extraterminal Domain Proteins, and WNT or MAPK Signaling, Inhibits c-MYC Expression and Proliferation of Colorectal Cancer Cells. Molecular cancer therapeutics.

[98] Nakamura Y, Hattori N, Iida N, Yamashita S, Mori A, Kimura K (2017). Targeting of super-enhancers and mutant BRAF can suppress growth of BRAF-mutant colon cancer cells via repression of MAPK signaling pathway. Cancer letters.

[99] Andricovich J, Perkail S, Kai Y, Casasanta N, Peng W, Tzatsos A (2018). Loss of KDM6A Activates Super-Enhancers to Induce Gender-Specific Squamous-like Pancreatic Cancer and Confers Sensitivity to BET Inhibitors. Cancer cell.

[100] Xie F, Huang M, Lin X, Liu C, Liu Z, Meng F (2018). The BET inhibitor I-BET762 inhibits pancreatic ductal adenocarcinoma cell proliferation and enhances the therapeutic effect of gemcitabine. Sci Rep.

[101] Amorim S, Stathis A, Gleeson M, Iyengar S, Magarotto V, Leleu X (2016). Bromodomain inhibitor OTX015 in patients with lymphoma or multiple myeloma: a dose-escalation, open-label, pharmacokinetic, phase 1 study. The Lancet Haematology.

[102] Stathis A, Zucca E, Bekradda M, Gomez-Roca C, Delord JP, de La Motte Rouge T (2016). Clinical Response of Carcinomas Harboring the BRD4-NUT Oncoprotein to the Targeted Bromodomain Inhibitor OTX015/MK-8628. Cancer discovery.

[103] Piquereau J, Boet A, Péchoux C, Antigny F, Lambert M, Gressette M (2019). The BET Bromodomain Inhibitor I-BET-151 Induces Structural and Functional Alterations of the Heart Mitochondria in Healthy Male Mice and Rats. International journal of molecular sciences.

[104] Faivre EJ, McDaniel KF, Albert DH, Mantena SR, Plotnik JP, Wilcox D (2020). Selective inhibition of the BD2 bromodomain of BET proteins in prostate cancer. Nature.

[105] Jiang F, Wei Q, Li H, Li H, Cui Y, Ma Y (2020). Discovery of novel small molecule induced selective degradation of the bromodomain and extra-terminal (BET) bromodomain protein BRD4 and BRD2 with cellular potencies. Bioorganic & medicinal chemistry.

[106] Wang W, Tang YA, Xiao Q, Lee WC, Cheng B, Niu Z (2021). Stromal induction of BRD4 phosphorylation Results in Chromatin Remodeling and BET inhibitor Resistance in Colorectal Cancer. Nature communications.

[107] Kelly AD, Issa JJ (2017). The promise of epigenetic therapy: reprogramming the cancer epigenome. Current opinion in genetics & development.

[108] Nilson KA, Guo J, Turek ME, Brogie JE, Delaney E, Luse DS (2015). THZ1 Reveals Roles for Cdk7 in Co-transcriptional Capping and Pausing. Molecular cell.

[109] Larochelle S, Amat R, Glover-Cutter K, Sansó M, Zhang C, Allen JJ (2012). Cyclin-dependent kinase control of the initiation-to-elongation switch of RNA polymerase II. Nature structural & molecular biology.

[110] Hu S, Marineau JJ, Rajagopal N, Hamman KB, Choi YJ, Schmidt DR (2019). Discovery and Characterization of SY-1365, a Selective, Covalent Inhibitor of CDK7. Cancer research.

[111] Jiang YY, Lin DC, Mayakonda A, Hazawa M, Ding LW, Chien WW (2017). Targeting super-enhancer-associated oncogenes in oesophageal squamous cell carcinoma. Gut.

[112] Huang CS, You X, Dai C, Xu QC, Li F, Wang L (2020). Targeting Super-Enhancers via Nanoparticle-Facilitated BRD4 and CDK7 Inhibitors Synergistically Suppresses Pancreatic Ductal Adenocarcinoma. Adv Sci (Weinh).

[113] Tsang FH, Law CT, Tang TC, Cheng CL, Chin DW, Tam WV (2019). Aberrant Super-Enhancer Landscape in Human Hepatocellular Carcinoma. Hepatology (Baltimore, Md).

[114] Zeng H, Yang H, Song Y, Fang D, Chen L, Zhao Z (2021). Transcriptional inhibition by CDK7/9 inhibitor SNS-032 suppresses tumor growth and metastasis in esophageal squamous cell carcinoma. Cell death & disease.

[115] Zhan T, Rindtorff N, Betge J, Ebert MP, Boutros M (2019). CRISPR/Cas9 for cancer research and therapy. Seminars in cancer biology.

[116] Cheng H, Dou X, Han JD (2016). Understanding super-enhancers. Sci China Life Sci.

[117] Zheng C, Liu M, Fan H (2020). Targeting complexes of super-enhancers is a promising strategy for cancer therapy. Oncology letters.

[118] Mill CP, Fiskus W, DiNardo CD, Qian Y, Raina K, Rajapakshe K (2019). RUNX1-targeted therapy for AML expressing somatic or germline mutation in RUNX1. Blood.

[119] Yoo KH, Hennighausen L, Shin HY (2019). Dissecting Tissue-Specific Super-Enhancers by Integrating Genome-Wide Analyses and CRISPR/Cas9 Genome Editing. Journal of mammary gland biology and neoplasia.

[120] Yu W, Chen K, Ye G, Wang S, Wang P, Li J (2021). SNP-adjacent super enhancer network mediates enhanced osteogenic differentiation of MSCs in ankylosing spondylitis. Human molecular genetics.

[121] Cong Z, Li Q, Yang Y, Guo X, Cui L, You T (2019). The SNP of rs6854845 suppresses transcription via the DNA looping structure alteration of super-enhancer in colon cells. Biochemical and biophysical research communications.

[122] Eid A, Alshareef S, Mahfouz MM (2018). CRISPR base editors: genome editing without double-stranded breaks. Biochem J.

[123] Li K, Liu Y, Cao H, Zhang Y, Gu Z, Liu X (2020). Interrogation of enhancer function by enhancer-targeting CRISPR epigenetic editing. Nature communications.

[124] Liu X, Zhang Y, Chen Y, Li M, Zhou F, Li K (2017). *In Situ* Capture of Chromatin Interactions by Biotinylated dCas9. Cell.

[125] Anzalone AV, Randolph PB, Davis JR, Sousa AA, Koblan LW, Levy JM (2019). Search-and-replace genome editing without double-strand breaks or donor DNA. Nature.

[126] Hu S, Marineau JJ Discovery and Characterization of SY-1365, a Selective, Covalent Inhibitor of CDK7. 2019; 79: 3479-91.

[127] Lin DC, Dinh HQ, Xie JJ, Mayakonda A, Silva TC, Jiang YY (2018). Identification of distinct mutational patterns and new driver genes in oesophageal squamous cell carcinomas and adenocarcinomas. Gut.

[128] Chen C, Maimaiti A, Zhang X, Qu H, Sun Q, He Q (2018). Knockdown of RAI14 suppresses the progression of gastric cancer. OncoTargets and therapy.

[129] Heyn H, Vidal E, Ferreira HJ, Vizoso M, Sayols S, Gomez A (2016). Epigenomic analysis detects aberrant super-enhancer DNA methylation in human cancer. Genome biology.

[130] Ke J, Tian J, Mei S, Ying P, Yang N, Wang X (2020). Genetic Predisposition to Colon and Rectal Adenocarcinoma Is Mediated by a Super-enhancer Polymorphism Coactivating CD9 and PLEKHG6. Cancer epidemiology, biomarkers & prevention: a publication of the American Association for Cancer Research, cosponsored by the American Society of Preventive Oncology.

[131] Lee D, Zhang MS, Tsang FH, Bao MH, Xu IM, Lai RK (2021). Adaptive and Constitutive Activations of Malic Enzymes Confer Liver Cancer Multilayered Protection Against Reactive Oxygen Species. Hepatology (Baltimore, Md).

[132] Xiong L, Wu F, Wu Q, Xu L, Cheung OK, Kang W (2019). Aberrant enhancer hypomethylation contributes to hepatic carcinogenesis through global transcriptional reprogramming. Nature communications.

[133] Han J, Meng J, Chen S, Wang X, Yin S, Zhang Q (2019). YY1 Complex Promotes Quaking Expression via Super-Enhancer Binding during EMT of Hepatocellular Carcinoma. Cancer research.

[134] Cui J, Liu J, Fan L, Zhu Y, Zhou B, Wang Y (2020). A zinc finger family protein, ZNF263, promotes hepatocellular carcinoma resistance to apoptosis via activation of ER stress-dependent autophagy. Translational oncology.

[135] Xiang X, Deng L, Xiong R, Xiao D, Chen Z, Yang F (2018). Tex10 is upregulated and promotes cancer stem cell properties and chemoresistance in hepatocellular carcinoma. Cell cycle (Georgetown, Tex).

[136] Lomberk G, Blum Y, Nicolle R, Nair A, Gaonkar KS, Marisa L (2018). Distinct epigenetic landscapes underlie the pathobiology of pancreatic cancer subtypes. Nature communications.

[137] Mathison AJ, Kerketta R, de Assuncao TM, Leverence E, Zeighami A, Urrutia G (2021). Kras(G12D) induces changes in chromatin territories that differentially impact early nuclear reprogramming in pancreatic cells. Genome biology.

[138] Barrett MT, Deiotte R, Lenkiewicz E, Malasi S, Holley T, Evers L (2017). Clinical study of genomic drivers in pancreatic ductal adenocarcinoma. British journal of cancer.

[139] Evan GI, Hah N, Littlewood TD, Sodir NM, Campos T, Downes M (2017). Re-engineering the Pancreas Tumor Microenvironment: A "Regenerative Program" Hacked. Clinical cancer research: an official journal of the American Association for Cancer Research.

[140] Hamdan FH, Johnsen SA (2018). DeltaNp63-dependent super enhancers define molecular identity in pancreatic cancer by an interconnected transcription factor network. Proceedings of the National Academy of Sciences of the United States of America.

